# Exploring the potential nutritional role of bioflavonoids in exercise rehabilitation: a kinematic perspective

**DOI:** 10.3389/fnut.2023.1221800

**Published:** 2023-06-30

**Authors:** Qiaoyin Tan, Bochao Chen, Cuicui Wu, Tianyi Shao

**Affiliations:** ^1^College of Education, Zhejiang Normal University, Jinhua, Zhejiang, China; ^2^College of Physical Education and Health Sciences, Zhejiang Normal University, Jinhua, Zhejiang, China

**Keywords:** potential nutritional role, bioflavonoids, exercise rehabilitation, kinematic perspective, bioflavonoids in exercise rehabilitation

## 1. Introduction

Bioflavonoids, as naturally derived polyphenolic compounds, have been studied extensively due to their potential health-promoting attributes (1). Their antioxidant, anti-inflammatory, and vasodilatory properties have been found to be instrumental in improving cardiovascular health, bolstering immune function, and reducing the risk of chronic diseases (2, 3). However, the potential implications of bioflavonoids within the realm of kinesiology and athletic rehabilitation have not been adequately explored. This review, therefore, aims to bridge this gap by providing an in-depth analysis of the nutritional mechanisms of bioflavonoids from a kinesiology standpoint, with an emphasis on their potential role in supporting athletic rehabilitation.

## 2. Subsections relevant for the subject

### 2.1. Modulation of inflammation and oxidative stress

Intense physical activity can induce inflammation and oxidative stress in athletes, which may lead to muscle damage and prolonged recovery periods (4). Bioflavonoids have been shown to modulate inflammation by inhibiting pro-inflammatory cytokines, such as tumor necrosis factor-alpha (TNF-α) and interleukin-6 (IL-6) (2). In addition, it also enhance antioxidant enzyme activity and keep reactive oxygen species (ROS) at low levels to avoid oxidative stress (3, 5). This may help reduce muscle damage and inflammation, thereby accelerating the recovery process in athletes.

### 2.2. Enhancing vascular function and perfusion

Bioflavonoids have been reported to improve vascular function by increasing nitric oxide (NO) production, a potent vasodilator, and reducing endothelial dysfunction (6). Enhanced vascular function can improve blood flow and oxygen delivery to the muscles (7), which may help speed up the removal of metabolic waste products and promote tissue repair (8). This may be particularly beneficial for athletes during the rehabilitation process, as improved blood flow can facilitate healing and optimize recovery (9).

### 2.3. Supporting collagen synthesis and connective tissue health

Collagen, a primary structural protein in connective tissues, plays a vital role in maintaining the integrity and strength of tendons, ligaments, and cartilage (10). Some bioflavonoids, such as proanthocyanidins, have been found to stimulate collagen synthesis and stabilize collagen structures by forming covalent cross-links (11). This may help support connective tissue health and function, potentially reducing the risk of injury and aiding in the rehabilitation of athletes recovering from musculoskeletal injuries.

### 2.4. Modulation of immune function

The immune system plays a crucial role in the body's response to injury and tissue repair. Intense exercise can temporarily suppress immune function, increasing the risk of infection and delaying the healing process (12, 13). Bioflavonoids have been shown to modulate immune function by enhancing the activity of natural killer cells, macrophages, and T-lymphocytes (14–16). This may help maintain a robust immune response during periods of intense training or injury (17), supporting the athlete's overall health and recovery.

### 2.5. Role in pain management

Pain is a common symptom associated with athletic injuries and can negatively impact the rehabilitation process (18). Some bioflavonoids, such as quercetin and hesperidin, have demonstrated analgesic properties (19) by inhibiting the synthesis and release of pro-inflammatory mediators, such as prostaglandins and leukotrienes, which play a role in pain perception (20, 21). Incorporating bioflavonoid-rich foods or supplements may provide a natural alternative for pain management during the athletic rehabilitation process (22), potentially reducing the need for pharmacological interventions.

### 2.6. Influence on energy metabolism

Energy metabolism is critical for athletes' performance and recovery. Bioflavonoids, such as quercetin (23), have been shown to influence energy metabolism by enhancing mitochondrial biogenesis and function (24). Improved mitochondrial function can increase energy production and efficiency, potentially supporting the athlete's recovery process and promoting a quicker return to optimal performance levels (25).

### 2.7. Potential synergistic effects with other nutrients

Bioflavonoids often coexist with other nutrients and phytochemicals in plant-based foods, which may lead to synergistic effects that can enhance their overall impact on athletic rehabilitation (26, 27). For example, combining bioflavonoids with other antioxidants, such as vitamins C and E, has been shown to enhance their antioxidant capacity and provide more significant protection against oxidative stress (28, 29). Additionally, bioflavonoids may interact with other nutrients, such as amino acids and fatty acids (30), to support tissue repair and recovery processes (31). Further research is needed to better understand these interactions and their implications for athletic rehabilitation.

## 3. Discussion

### 3.1. Consideration of bioflavonoid sources and dietary patterns

While bioflavonoids are present in many plant-based foods, the specific types and concentrations of these compounds can vary greatly among different sources (32). Moreover, the bioavailability and effectiveness of bioflavonoids can be influenced by factors such as food processing, cooking methods, and the presence of other dietary components (33, 34). Future research should investigate the most effective sources and dietary patterns for athletes, taking into account the bioflavonoid content and overall nutrient profile of different foods and supplements (35). We made a recommended table for daily consumption of flavonoids, as shown in Table 1.

**Table 1:** Recommended table for daily consumption of flavonoids.

**Types of bioflavonoids and their food sources**	**Recommended daily intake of bioflavonoids for athletes**	**Potential health benefits of bioflavonoids for athletes**	**Potential side effects or interactions with medications or other supplements**	**Current evidence and recommendations for incorporating bioflavonoids into athletic rehabilitation plans**
Flavonols: quercetin, kaempferol, myricetin	Fruits (apples, grapes, berries, citrus fruits), vegetables (onions, kale, broccoli), tea	Recommended intake of flavonols ranges from 50–500 mg/day	Lowers inflammation and oxidative stress, improves vascular function, enhances immune function, manages pain, influences energy metabolism	Possible interactions with medications that affect blood clotting (e.g., aspirin, warfarin)
Flavanones: hesperidin, naringenin	Citrus fruits (oranges, grapefruits), tomatoes	Recommended intake of hesperidin ranges from 25–500 mg/day	Lowers inflammation and oxidative stress, improves vascular function, enhances immune function, manages pain	Possible interactions with medications that affect blood pressure (e.g., calcium channel blockers)
Flavones: apigenin, luteolin	Parsley, celery, chamomile tea	Recommended intake of apigenin ranges from 5–50 mg/day	Lowers inflammation and oxidative stress, improves vascular function, enhances immune function, manages pain	Not known to have significant side effects or interactions
Flavanols: catechins, epicatechins	Tea, cocoa, berries, grapes, nuts	Recommended intake of catechins ranges from 100–1,000 mg/day	Lowers inflammation and oxidative stress, improves vascular function, enhances immune function, manages pain	Possible interactions with medications that affect blood pressure (e.g., calcium channel blockers)
Anthocyanins: cyanidin, delphinidin	Berries, grapes, cherries, pomegranates	Not established, but recommended intake of anthocyanins ranges from 50–1,000 mg/day	Lowers inflammation and oxidative stress, improves vascular function, enhances immune function, manages pain	Not known to have significant side effects or interactions
Isoflavones: genistein, daidzein	Soybeans, soy products	Recommended intake of isoflavones ranges from 50–150 mg/day	Lowers inflammation and oxidative stress, improves vascular function, enhances immune function	Possible interactions with medications that affect hormones (e.g., birth control pills, hormone replacement therapy)

### 3.2. Integrating bioflavonoids into comprehensive rehabilitation strategies

The current understanding of bioflavonoids' nutritional mechanisms from a kinesiology perspective suggests that these compounds may have potential applications in supporting athletic rehabilitation (30, 36). Their ability to modulate inflammation, oxidative stress, vascular function, immune function, and energy metabolism, as well as their potential synergistic effects with other nutrients, may contribute to improved recovery outcomes for athletes (1–3, 9, 24). But it is essential to recognize that bioflavonoids should not be considered a panacea for athletic rehabilitation. While these compounds may have potential benefits, they should be viewed as a complementary approach within a comprehensive rehabilitation strategy. Such strategies should encompass a multidisciplinary approach, including physical therapy, strength and conditioning, psychological support, and individualized nutrition plans (37). By integrating bioflavonoid-rich foods or supplements into these comprehensive strategies, athletes may be better positioned to optimize their recovery and return to peak performance levels.

### 3.3. Educational and practical implications for practitioners

As the understanding of bioflavonoids' nutritional mechanisms and their potential role in athletic rehabilitation grows, it is crucial for practitioners working with athletes, such as sports dietitians, physiotherapists, and strength and conditioning coaches, to stay informed about the latest evidence and practical applications (38). This will enable them to educate athletes on the potential benefits of bioflavonoids and guide them in incorporating these compounds into their rehabilitation plans. Additionally, practitioners should be aware of potential interactions between bioflavonoids and medications or other supplements, as well as any contraindications or potential side effects that may arise.

However, several knowledge gaps and limitations need to be addressed to fully understand and optimize the use of bioflavonoids in athletic rehabilitation. For instance, more research is needed to determine the optimal dosages, bioavailability, and efficacy of various bioflavonoids for different types of athletic injuries and rehabilitation needs (39). Additionally, understanding the influence of individual genetic variations and the role of the gut microbiota in bioflavonoid metabolism and activity may help personalize nutrition strategies for athletes (40). This review has offered a novel perspective on the role of bioflavonoids in athletic rehabilitation, setting the stage for further research in this domain. The instructional contribution of this work lies in the potential guidance it offers to practitioners working with athletes, enabling them to incorporate bioflavonoids more effectively into their rehabilitation strategies.

In conclusion, an improved understanding of the nutritional mechanisms of bioflavonoids from a kinesiology perspective holds promise for their potential application in athletic rehabilitation. While evidence suggests that these compounds may have a variety of beneficial effects, further research is needed to elucidate their specific mechanisms, identify optimal sources and dosages, and evaluate their efficacy in well-designed clinical trials (41). While bioflavonoids' health benefits have been widely recognized, their role in mitigating inflammation and oxidative stress, particularly within the scope of kinesiology, remains understudied. Our research fills this gap, extending the understanding of bioflavonoids to the domain of athletic rehabilitation. Future research should focus on elucidating the specific cellular and molecular mechanisms underlying their diverse biological activities, evaluating their efficacy in well-designed clinical trials, and investigating the complex interactions between bioflavonoids and other dietary components, genetic factors, and gut microbiota. By deepening our understanding of bioflavonoids' nutritional mechanisms and their interactions with other dietary components, genetic factors, and the gut microbiota, we can develop evidence-based strategies to support athletes' recovery and overall health. It is essential for practitioners working with athletes to stay informed about the latest evidence and practical applications of bioflavonoids and integrate them into comprehensive, multidisciplinary rehabilitation strategies.

## Author contributions

QT wrote the first draft. BC was responsible for collecting data. CW was responsible for checking the format. TS was responsible for conceiving ideas. All authors contributed to the article and approved the submitted version.
